# Ai-Fen Solid Dispersions: Preparation, Characterization, and Enhanced Therapeutic Efficacy in a Rat Model of Oral Ulceration

**DOI:** 10.3390/ph19010007

**Published:** 2025-12-19

**Authors:** Bing-Nan Liu, Kai-Lang Mu, Chang-Liu Shao, Ping-Xuan Xie, Jun-Li Xie, Mei-Hui He, Yu-Chen Liu, Ke Zhong, Yuan Yuan, Xiao-Min Tang, Yu-Xin Pang

**Affiliations:** 1 College of Pharmacy, Guizhou University of Traditional Chinese Medicine, Guiyang 550025, China; lbn0714@163.com (B.-N.L.); mkl980818@163.com (K.-L.M.); shaochangliu178@163.com (C.-L.S.); xiepingxuan@stu.gzy.edu.cn (P.-X.X.); xiejunli1008@163.com (J.-L.X.); hmh20040329@163.com (M.-H.H.); lyc8564732@163.com (Y.-C.L.); lionzhongke@163.com (K.Z.); 2 College of Life Sciences and Biopharmaceuticals, Guangdong Pharmaceutical University, Guangzhou 510000, China; yuanyuanhaida@126.com; 3 College of Traditional Chinese Medicine, Guangdong Pharmaceutical University, Guangzhou 510000, China; txm1209@163.com

**Keywords:** Ai-Fen, solid dispersion, levoborneol, recurrent oral ulcer, bioavailability enhancement, oral mucosal repair

## Abstract

**Background/Objectives:** Recurrent oral ulceration (ROU) is the most prevalent disorder of the oral mucosa, affecting approximately 20% of the global population. Current therapies are limited by adverse effects and high recurrence rates. Ai-Fen, enriched in the anti-inflammatory monoterpenoid L-borneol (54.3% *w*/*w*), exhibits therapeutic potential but suffers from poor aqueous solubility and low bioavailability. This study aimed to improve the physicochemical properties and in vivo efficacy of Ai-Fen through the preparation of solid dispersions. **Methods:** Ai-Fen solid dispersions (AF-SD) were prepared by a melt-fusion method using polyethylene glycol 6000 (PEG 6000) as the carrier. An L_9_(3^3^) orthogonal design was employed to optimize three critical parameters: drug-to-carrier ratio, melting temperature, and melting duration. The resulting dispersions were systematically characterized by differential scanning calorimetry (DSC), powder X-ray diffraction (PXRD), scanning electron microscopy (SEM), and Fourier-transform infrared spectroscopy (FTIR). A chemically induced ROU model in rats (*n* = 8 per group) was established to evaluate the effects of AF-SD on ulcer area, serum inflammatory cytokines (TNF-α, IL-6), vascular endothelial growth factor (VEGF) levels, and histopathological outcomes. **Results:** The optimal formulation was obtained at a drug-to-carrier ratio of 1:2, a melting temperature of 70 °C, and a melting time of 5 min. Under these conditions, L-borneol release increased 2.5-fold. DSC and PXRD confirmed complete conversion of Ai-Fen to an amorphous state, while FTIR revealed a 13 cm^−1^ red shift in the O-H stretching band, indicating hydrogen-bond formation. In vivo, AF-SD reduced ulcer area by 60.7% (*p* < 0.001) and achieved a healing rate of 74.16%. Serum TNF-α and IL-6 decreased by 55.5% and 49.6%, respectively (both *p* < 0.001), whereas VEGF increased by 89.6% (*p* < 0.001). Histological analysis confirmed marked reduction in inflammatory infiltration, accelerated re-epithelialization (score 2.50), and a 5.9-fold increase in neovascularization. **Conclusions:** AF-SD markedly enhanced the bioavailability of Ai-Fen through amorphization and accelerated ROU healing, likely via dual mechanisms involving suppression of nuclear factor kappa-B (NF-κB)-mediated inflammation and promotion of angiogenesis. This formulation strategy provides a promising approach for modernizing traditional herbal medicines.

## 1. Introduction

Recurrent oral ulceration (ROU), also referred to as recurrent aphthous stomatitis (RAS), is one of the most common disorders of the oral mucosa. Its global prevalence is approximately 20%, and it can reach as high as 60% in certain populations [1,2]. Clinically, ROU is characterized by recurrent, painful ulcerations of the oral mucosa, typically presenting as round or oval superficial lesions accompanied by a pronounced burning sensation. These symptoms significantly impair eating, speaking, and overall quality of life [3]. Although individual episodes usually resolve spontaneously within 1–2 weeks, the high relapse rate—up to 40–50% annually—and periodic pain impose a substantial and persistent burden on affected individuals. The etiology of ROU remains incompletely understood. Current evidence suggests that its occurrence is multifactorial, involving immune dysregulation, genetic susceptibility, oxidative stress, microbial imbalance, and local trauma [4,5,6,7]. Among these, aberrant inflammatory responses are believed to play a central pathogenic role. Elevated levels of pro-inflammatory cytokines—such as tumor necrosis factor-α (TNF-α) and interleukin-6 (IL-6)—have been consistently detected in ulcerative tissues, whereas anti-inflammatory mediators and pro-repair factors are insufficiently expressed [8,9]. Recent studies have further elucidated that the imbalance between Th17 and Treg cells plays a pivotal role in the chronicity of ROU, highlighting the need for therapies that simultaneously modulate inflammation and promote tissue repair [10,11].

Existing therapeutic strategies for ROU primarily include topical corticosteroids, antimicrobial agents, immunomodulatory drugs, and various physical therapies [12,13]. However, each approach presents notable limitations. Corticosteroids offer rapid symptomatic relief but may induce adverse effects such as oral fungal infections and mucosal atrophy with prolonged use. Antimicrobials provide limited benefit for noninfectious ulcers and may disrupt the microbial ecosystem. Immunomodulators such as thalidomide have demonstrated efficacy, yet their clinical use is restricted by teratogenicity and neurotoxicity. Moreover, many available drugs have inherently low bioavailability, short residence times on the mucosal surface, and require frequent administration, leading to poor patient adherence. Consequently, there is a pressing need for novel ROU therapies that are effective, safe, have minimal adverse effects, and possess clear mechanisms of action.

Natural products have attracted increasing attention in the management of ROU owing to their multi-target activities, favorable safety profiles, and low cost [14,15,16]. Ai-Fen, a traditional medicinal material derived from the leaves of *Blumea balsamifera* (L.) DC., has been used for centuries in the ethnomedicine of southwestern China to treat oral ulcers, sore throat, traumatic injuries, and other inflammatory conditions. Historically regarded as an intermediate product in the extraction of natural borneol, Ai-Fen itself has recently been recognized for its intrinsic pharmacological value [17,18]. Phytochemical analyses reveal that Ai-Fen contains 40–60% (*w*/*w*) L-borneol, along with β-caryophyllene, camphor, and other terpenoids. Among these constituents, L-borneol is the principal active compound and has been extensively reported to exert anti-inflammatory, antimicrobial, antioxidant, and penetration-enhancing effects [19,20]. Although Ai-Fen is widely used in TCM clinical practice for throat and oral ailments, its application is largely restricted to coarse powders or simple ethanolic extracts. Previous pharmacological studies have established that while the crude extract possesses potent anti-inflammatory activity, its efficacy is often inconsistent due to the sublimation of borneol and poor wettability in the aqueous oral environment [21]. Furthermore, clinical feedback suggests that direct application of the raw powder can cause a gritty sensation and has a short retention time on the mucosa, necessitating frequent administration. Therefore, developing a formulation that simultaneously enhances solubility, stability, and patient compliance is critical for unlocking the full therapeutic potential of Ai-Fen.

L-borneol has been shown to inhibit NF-κB signaling, thereby reducing the expression of TNF-α, IL-1β, and IL-6, while upregulating the anti-inflammatory cytokine IL-10 [22,23]. Microbiological studies further demonstrate that L-borneol exhibits marked inhibitory activity against common oral pathogens, including Staphylococcus aureus, Escherichia coli, and Candida albicans, with minimum inhibitory concentrations (MICs) ranging from 0.125 to 2.0 mg/mL [24]. In addition, its traditional role as a “channel-opening” agent enhances the bioavailability of co-administered drugs, a property widely applied in Chinese medicinal formulations. Our previous work has confirmed the strong inhibitory effects of Ai-Fen on oral anaerobes, particularly *Fusobacterium nucleatum*. Despite its therapeutic potential, the clinical translation of Ai-Fen is hampered by a critical limitation: its extremely poor aqueous solubility (<0.5 mg/mL at 25 °C) [20]. As a hydrophobic terpenoid-rich extract, Ai-Fen exhibits limited dissolution following oral or topical administration, leading to insufficient bioavailability and subtherapeutic drug concentrations at ulcerative sites [25]. This bottleneck severely restricts its development into modern clinical formulations.

To address the low bioavailability of poorly soluble drugs, contemporary pharmaceutical sciences have developed several solubilization strategies, including nanocrystals, liposomes, cyclodextrin inclusion complexes, and solid dispersions. Among these approaches, solid dispersion technology has emerged as one of the most effective and versatile methods due to its simplicity, scalability, cost efficiency, and favorable stability profile. Solid dispersions incorporate the drug in a molecular, amorphous, or microcrystalline state within an inert carrier matrix [26,27,28]. Their enhanced solubility is achieved through multiple mechanisms: (1) transformation of the crystalline drug into an amorphous form with higher free energy; (2) reduction in drug particle size to the micro- or nanometer scale; (3) improved wettability via hydrophilic carriers; and (4) suppression of drug recrystallization during dissolution [29,30,31].

Polyethylene glycol (PEG) is one of the most widely used carriers for solid dispersions because of its excellent water solubility, biocompatibility, chemical stability, and well-established pharmaceutical safety. PEG 6000, with a melting point of 58–63 °C and suitable viscosity, is particularly advantageous for melt-fusion methods and provides a stable matrix that minimizes drug recrystallization during storage [32,33]. For oral mucosal delivery, PEG-based solid dispersions offer additional benefits. Their hydrophilic matrix rapidly swells and disintegrates in saliva, facilitating prompt drug release and uniform distribution across ulcer surfaces while prolonging mucosal contact [34,35]. PEG also exhibits mild mucosal protective and lubricating effects that help alleviate pain at ulcer sites. Recent studies have successfully employed PEG-based solid dispersions for natural compounds such as curcumin, resveratrol, and quercetin in the treatment of oral inflammatory diseases, demonstrating improved dissolution and enhanced therapeutic efficacy [36,37,38,39]. Although other solubilization strategies such as cyclodextrin inclusion complexes and liposomes have been reported for borneol-related compounds, they are often limited by low drug loading capacity, instability during storage, or high manufacturing costs [40]. In contrast, solid dispersion technology, particularly the solvent-free melt-fusion method, offers a superior balance of high drug loading, industrial scalability, and cost-effectiveness. To date, no research has systematically explored the formulation of Ai-Fen into solid dispersions to enhance its bioavailability or systematically evaluated its anti-ROU activity.

Against this backdrop, the present study aimed to overcome the solubility and bioavailability limitations of Ai-Fen by developing a PEG 6000–based solid dispersion and to investigate its therapeutic effects and underlying mechanisms in an ROU model. Specifically, Ai-Fen solid dispersions were prepared using a melt-fusion method, and process parameters—including drug-to-carrier ratio, melting temperature, and melting time—were optimized through single-factor experiments and an orthogonal design. The physicochemical properties of the optimized formulation were analyzed by differential scanning calorimetry (DSC), powder X-ray diffraction (PXRD), scanning electron microscopy (SEM), and Fourier-transform infrared spectroscopy (FTIR) to elucidate the solid-state behavior of Ai-Fen and its interactions with the carrier. Finally, a chemically induced rat ROU model was established to evaluate the therapeutic potential of the formulation through assessments of ulcer healing kinetics, inflammatory cytokine profiles, angiogenesis markers, and histopathological features. The resulting solid dispersion demonstrates promising translational potential and may offer an effective, economical, and low-toxicity therapeutic option for patients with ROU.

## 2. Results

### 2.1. Optimization of Ai-Fen Solid Dispersion Preparation

#### 2.1.1. Single-Factor Experiments

To identify the key preparation parameters influencing the dissolution behavior of the Ai-Fen solid dispersions, the effects of drug-to-carrier ratio, melting temperature, and melting time on the cumulative release of L-borneol were systematically examined.

(1) Effect of drug-to-carrier ratio

**Figure 1:**
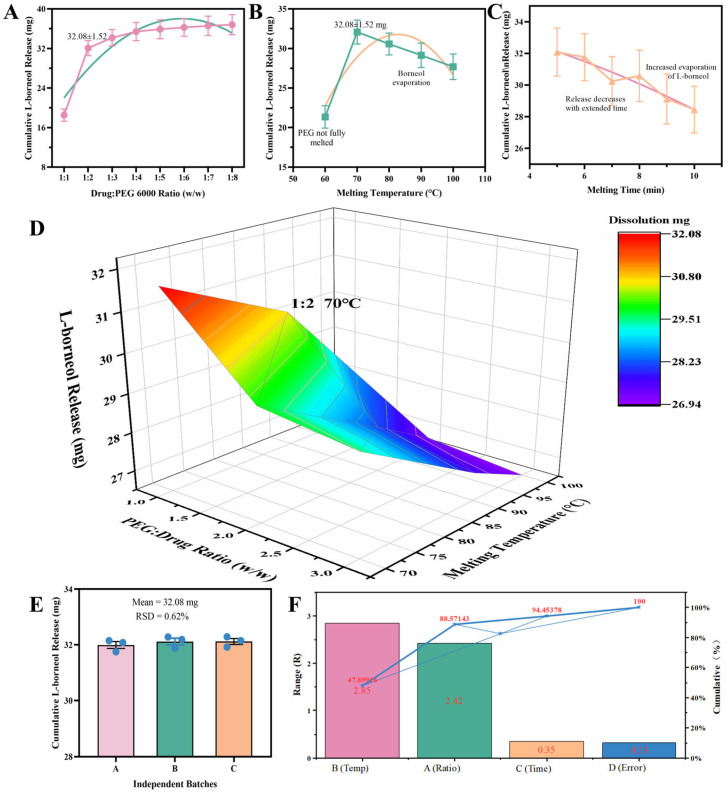
Optimization and validation of AF-SD preparation parameters. (**A**) Effect of drug-to-carrier ratio on L-borneol release. (**B**) Effect of melting temperature. (**C**) Effect of melting duration. (**D**) 3D response surface of factor interactions. (**E**) Batch-to-batch reproducibility under optimal conditions. (**F**) ANOVA and contribution analysis for the orthogonal design.

As shown in Figure 1A, under fixed conditions of 70 °C and 5 min, the cumulative release of L-borneol at 25 min increased initially and then reached a plateau as the proportion of PEG 6000 increased (drug-to-carrier ratios from 1:1 to 1:8). At a ratio of 1:1, the cumulative release was only 18.52 ± 1.24 mg. Increasing the carrier to a ratio of 1:2 significantly enhanced release to 32.08 ± 1.52 mg (*p* < 0.01). Further increases to 1:3 and 1:4 yielded 34.17 ± 1.68 mg and 35.42 ± 1.85 mg, respectively, but with diminishing improvement. Ratios of 1:6 and above resulted in release values stabilizing at 35–37 mg with no significant differences. Considering dissolution efficiency and material cost, the preliminary optimal ratio ranged from 1:2 to 1:6.

(2) Effect of melting temperature

Under a fixed drug-to-carrier ratio of 1:2 and melting time of 5 min, melting temperature had a pronounced effect on dissolution (Figure 1B). At 60 °C, incomplete melting of PEG 6000 resulted in poor dispersion of Ai-Fen and a low cumulative release (21.35 ± 1.42 mg). Raising the temperature to 70 °C markedly increased release to 32.08 ± 1.52 mg. However, further increases to 80 °C, 90 °C, and 100 °C led to progressively lower release values of 30.57 ± 1.38 mg, 29.14 ± 1.55 mg, and 27.68 ± 1.62 mg, respectively. This decline was likely due to volatilization losses of L-borneol at elevated temperatures. Therefore, 70 °C was identified as the optimal melting temperature.

(3) Effect of melting time

At a fixed drug-to-carrier ratio of 1:2 and temperature of 70 °C, extending the melting time from 5 to 10 min caused the cumulative release to gradually decline from 32.08 ± 1.52 mg to 28.45 ± 1.48 mg (Figure 1C). Longer heating did not improve dissolution; instead, prolonged exposure likely exacerbated volatilization of the active component. Thus, 5 min was determined to be the optimal melting duration.

Based on the single-factor results, the preliminary optimal preparation window was determined as: drug-to-carrier ratio 1:2–1:6, melting temperature 70–90 °C, and melting time 5–7 min.

#### 2.1.2. Orthogonal Design Optimization

An L_9_(3^3^) orthogonal design was employed to further refine the preparation parameters. The experimental conditions and corresponding dissolution results are summarized in Table 1 and Table 2. Range analysis revealed that the influence of each factor on cumulative L-borneol release followed the order: melting temperature (B) > drug-to-carrier ratio (A) > melting time (C). The optimal combination determined by the orthogonal design was A_1_B_1_C_2_, corresponding to a 1:2 ratio, 70 °C melting temperature, and 6 min melting time. ANOVA confirmed that both the drug-to-carrier ratio and melting temperature significantly affected dissolution, whereas melting time had no significant effect (Figure 1D,F). Integrating range analysis and ANOVA results, the final optimized preparation conditions were established as 1:2 drug-to-carrier ratio, 70 °C melting temperature, and 5 min melting time. The shorter duration (5 min instead of 6 min) was selected to reduce processing time and minimize volatilization loss.

#### 2.1.3. Validation Experiment

To verify the reliability of the optimized preparation conditions, three independent batches of AF-SD were prepared using the final optimal parameters (ratio 1:2, 70 °C, 5 min). The cumulative release of L-borneol at 25 min for the three batches was 31.85 mg, 32.24 mg, and 32.15 mg, respectively, with a mean value of 32.08 ± 0.20 mg and a relative standard deviation (RSD) of 0.62% (Figure 1E). These results demonstrated excellent reproducibility, confirming the feasibility of the optimized process for subsequent experiments.

### 2.2. Physicochemical Characterization of the Solid Dispersion

#### 2.2.1. Dissolution Behavior

**Figure 2:**
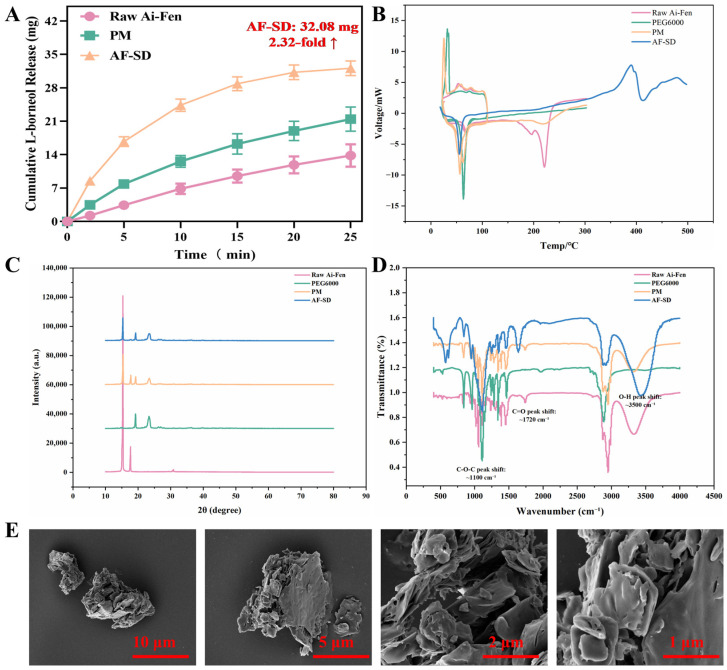
Multidimensional physicochemical characterization of AF-SD confirming amorphization. (**A**) Dissolution profiles. (**B**) Differential scanning calorimetry (DSC) thermograms. (**C**) Powder X-ray diffraction (PXRD) patterns. (**D**) Fourier transform infrared (FTIR) spectra. (**E**) Scanning electron microscopy (SEM) images.

The in vitro dissolution profiles of the optimized AF-SD, raw Ai-Fen, and the physical mixture (PM) are shown in Figure 2A. Raw Ai-Fen exhibited a very slow dissolution rate, with a cumulative release of only 13.82 ± 0.85 mg within 30 min, corresponding to approximately 12.5% cumulative release (calculated based on an L-borneol content of 54.3%). The PM showed slightly improved dissolution, with a cumulative release of 21.45 ± 1.25 mg at 30 min, but the enhancement remained limited. In contrast, AF-SD displayed markedly enhanced dissolution performance. It released 16.74 ± 1.02 mg of L-borneol within the first 5 min, followed by a sustained increase to 24.36 ± 1.28 mg at 10 min and 32.08 ± 1.52 mg (29.0%) at 25 min. Compared with the raw material, the cumulative release of L-borneol from AF-SD at 25 min was increased by 2.5-fold (*p* < 0.001), with a corresponding 2.5-fold increase in cumulative release percentage.

#### 2.2.2. Differential Scanning Calorimetry (DSC)

DSC thermograms of Ai-Fen, PEG 6000, PM, and AF-SD are presented in Figure 2B. Raw Ai-Fen exhibited a sharp endothermic peak at 204.6 °C with a melting enthalpy (ΔHm) of 92.5 J/g, corresponding to the melting of crystalline L-borneol and indicating that Ai-Fen existed in a crystalline state. PEG 6000 displayed a characteristic melting peak at 60.8 °C with a ΔHm of 168.3 J/g. The PM thermogram showed two distinct endothermic peaks at 61.2 °C (PEG 6000) and 203.8 °C (Ai-Fen), with peak positions and shapes closely resembling those of the individual components and only reduced intensity according to the mixing ratio, suggesting that no significant interaction occurred during simple physical mixing.

In striking contrast, the characteristic melting peak of Ai-Fen at 204.6 °C completely disappeared in the AF-SD thermogram. Only the PEG 6000 melting peak remained, appearing at 61.5 °C with slight peak broadening and a minor shift toward lower temperature (~0.7 °C). In addition, the experimental melting enthalpy of AF-SD (133.2 J/g) was lower than the theoretical value (143.0 J/g, calculated for a 1:2 ratio), representing a reduction of approximately 6.9%. These findings indicate that Ai-Fen in AF-SD was converted from a crystalline to an amorphous state and was highly dispersed within the PEG 6000 matrix, with possible intermolecular interactions between the drug and the carrier. Additionally, the broad endothermic event observed around 350 °C in the AF-SD thermogram corresponds to the thermal decomposition of the polymeric carrier, PEG 6000, rather than a phase transition of the drug.

#### 2.2.3. Powder X-Ray Diffraction (PXRD)

PXRD patterns of Ai-Fen, PEG 6000, PM, and AF-SD are shown in Figure 2C. Raw Ai-Fen exhibited sharp diffraction peaks at 2θ = 14.2° and 17.5°, indicative of a highly ordered crystalline structure. PEG 6000 showed two major diffraction peaks at 2θ = 19.3° and 23.5°, characteristic of a semicrystalline polymer. The PXRD pattern of the PM was essentially a superposition of the individual patterns of Ai-Fen and PEG 6000; diffraction peaks at 2θ = 14.2°, 17.5°, 19.3°, and 23.5° were all present, with intensities reduced according to the component proportions.

In contrast, The characteristic diffraction peaks of Ai-Fen at 2θ = 17.5° disappeared, while the peak at 14.2° was drastically reduced to a negligible intensity in the AF-SD pattern. Only the PEG 6000 peaks at 2θ = 19.3° and 23.5° were retained, with slightly reduced intensity and mild peak broadening. These observations are consistent with the DSC findings and suggest that Ai-Fen in AF-SD has predominantly converted to an amorphous form, although trace microcrystallinity may remain.

#### 2.2.4. Fourier Transform Infrared Spectroscopy (FTIR)

FTIR spectra of Ai-Fen, PEG 6000, PM, and AF-SD are shown in Figure 2D. The characteristic absorption bands of raw Ai-Fen included: 3323 cm^−1^ (O–H stretching), 2952 and 2870 cm^−1^ (C–H stretching), 1637 cm^−1^ (C=O stretching), 1456 cm^−1^ (C–H bending), and 1038 cm^−1^ (C–O stretching). PEG 6000 displayed major absorption bands at 2890 cm^−1^ (C–H stretching), 1467 cm^−1^ (C–H bending), 1342 cm^−1^ (C–H rocking), 1108 cm^−1^ (C–O–C ether stretching), and 950 cm^−1^ (C–H out-of-plane bending). The FTIR spectrum of the PM was essentially a simple combination of the Ai-Fen and PEG 6000 spectra, with no obvious shifts in band positions or changes in relative intensity.

By contrast, the AF-SD spectrum exhibited clear evidence of intermolecular interactions. The O-H stretching band of Ai-Fen shifted from 3323 to 3310 cm^−1^ (Δν = 13 cm^−1^) and became broader, suggesting the formation of new hydrogen bonds. The C=O stretching band of Ai-Fen also shifted slightly from 1637 to 1631 cm^−1^ (Δν = 6 cm^−1^), indicating possible involvement of carbonyl groups in hydrogen-bond formation. Meanwhile, the C–O–C ether stretching band of PEG 6000 shifted from 1108 to 1113 cm^−1^ (Δν = 5 cm^−1^), with a slight increase in intensity. Other characteristic peaks of Ai-Fen (2952, 2870, 1456, 1038 cm^−1^) were retained in the AF-SD spectrum but showed reduced intensity, consistent with the theoretical expectations for a 1:2 drug-to-carrier ratio. Taken together, these spectral changes indicate that Ai-Fen and PEG 6000 form a stable solid dispersion through hydrogen-bonding and related intermolecular interactions. Such interactions help suppress drug recrystallization and maintain the stability of the amorphous state.

#### 2.2.5. Scanning Electron Microscopy (SEM)

Representative SEM images of Ai-Fen, PEG 6000, PM, and AF-SD at different magnifications are shown in Figure 2E. At 2000× magnification (Figure 2E), raw Ai-Fen appeared as irregular block-like and needle-like crystals with smooth surfaces and sharp edges, with particle sizes in the range of 5–20 μm, characteristic of crystalline materials. PEG 6000 appeared as larger irregular block-like particles with relatively smooth surfaces and particle sizes of 20–50 μm. The PM images showed simple physical aggregation of Ai-Fen crystals and PEG particles, with clearly distinguishable morphologies and well-defined interfaces, indicating the absence of significant interactions between the two components.

In striking contrast, AF-SD exhibited a completely different microstructure. At 2000× magnification, the regular crystalline features of Ai-Fen had completely disappeared, and the sample surface appeared as a uniform amorphous matrix without discernible individual drug crystals. At 5000× magnification, AF-SD displayed a smooth, compact matrix with only occasional small pores (<1 μm), but overall homogeneous morphology. These morphological changes corroborate the DSC and PXRD results and provide direct microstructural evidence that Ai-Fen is highly dispersed in an amorphous form within the PEG 6000 matrix.

### 2.3. Effect of AF-SD on Oral Ulcer Healing

#### 2.3.1. Effect on Ulcer Area

**Figure 3:**
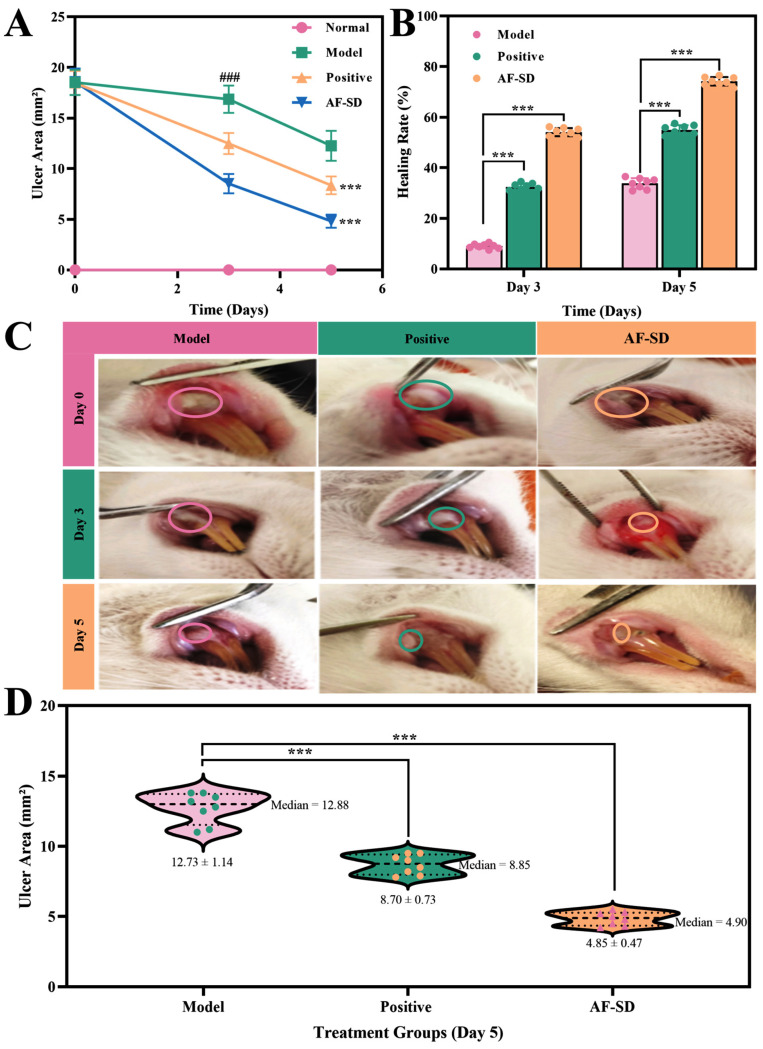
Therapeutic effects of AF-SD on healing of recurrent oral ulcers in rats. (**A**) Time-course changes in ulcer area. (**B**) Comparison of healing rates on days 3 and 5. (**C**) Representative macroscopic photographs of the healing process. (**D**) Violin plot of ulcer area distribution on day 5. *** indicates *p* < 0.001, ^###^ indicates that there is a significant difference between the control group and the control group.

Changes in ulcer area across the four experimental groups at different time points are shown in Figure 3A,D. At 24 h after ulcer induction (prior to treatment, day 0), no significant differences were observed among groups, confirming successful model establishment and comparable baseline ulcer sizes.

By day 3 of treatment, the ulcer area in the model group showed a slight reduction to 16.85 ± 1.35 mm^2^, reflecting a natural healing trend. The positive control group exhibited a greater reduction to 12.48 ± 1.05 mm^2^. Notably, the AF-SD group showed a significant decrease to 8.52 ± 0.95 mm^2^, comparable to the positive control but without a significant difference between the two groups, indicating a therapeutic effect equivalent to that of the clinically used spray.

By day 5, ulcer area in the model group had further decreased to 12.26 ± 1.48 mm^2^. The positive control group showed a continued reduction to 8.35 ± 0.88 mm^2^, whereas the AF-SD group displayed a markedly smaller ulcer area of 4.82 ± 0.65 mm^2^, which was significantly lower than that of the positive control (*p* < 0.001). These findings suggest that AF-SD exerted a superior healing effect compared with the positive control on day 5.

Representative macroscopic images are shown in Figure 3C. In the AF-SD group, the ulcer margins began to blur on day 3, accompanied by reduced mucosal congestion. By day 5, ulcer size was markedly diminished and the ulcer bed appeared pinkish, indicating favorable epithelial regeneration.

#### 2.3.2. Analysis of Ulcer Healing Rate

To further illustrate the therapeutic efficacy of each treatment, ulcer healing rates on days 3 and 5 were calculated, as shown in Figure 3B. On day 3, the healing rate of the model group was 9 ± 1.2%, while the positive control group reached 32.35 ± 2.1%. The AF-SD group showed a significantly higher healing rate of 54.08 ± 3.8%, which was markedly superior to that of the positive control (*p* < 0.001).

On day 5, the healing rate increased to 33.8 ± 6.5% in the model group. The positive control group exhibited a healing rate of 54.9 ± 5.3% (*** *p* < 0.001 vs. model group), whereas the AF-SD group achieved the highest healing rate of 74.16 ± 4.9% (*** *p* < 0.001 vs. model group). Collectively, these results demonstrate that AF-SD significantly accelerated ulcer healing. Its therapeutic advantage became evident as early as day 3, surpassing the positive control, and by day 5, AF-SD maintained healing efficacy comparable to or better than that of the positive control.

### 2.4. Effect of AF-SD on Inflammatory Cytokines and VEGF

#### 2.4.1. Serum Levels of TNF-α and IL-6

**Figure 4:**
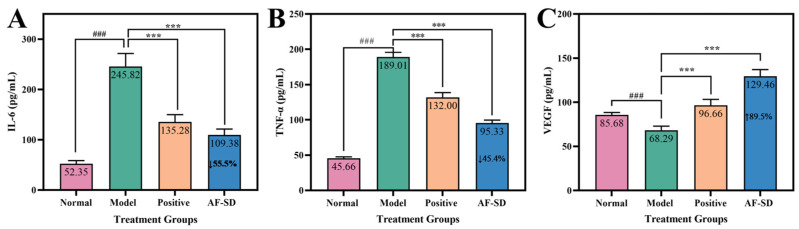
Effects of AF-SD on serum inflammatory cytokines and angiogenic factors. (**A**) Interleukin-6 (IL-6). (**B**) Tumor necrosis factor-α (TNF-α). (**C**) Vascular endothelial growth factor (VEGF). *** indicates *p* < 0.001, ^###^ indicates that there is a significant difference between the control group and the control group.

As shown in Figure 4A, the serum TNF-α concentration in the normal group was 52.35 ± 3.8 pg/mL. In contrast, the model group exhibited a dramatic increase to 245.82 ± 9.2 pg/mL (^###^ *p* < 0.001 vs. normal), confirming that ulcer induction triggered a pronounced systemic inflammatory response. Treatment with the positive control reduced TNF-α levels to 135.28 ± 7.5 pg/mL (*** *p* < 0.001 vs. model), representing a 44.9% decrease. AF-SD further reduced TNF-α to 109.38 ± 6.8 pg/mL (*** *p* < 0.001 vs. model), corresponding to a 55.5% reduction, which was significantly lower than that observed in the positive control group.

A similar trend was observed for IL-6 (Figure 4B). Serum IL-6 levels were 45.66 ± 3.5 pg/mL in the normal group and markedly increased to 189.01 ± 11.6 pg/mL in the model group (^###^ *p* < 0.0001 vs. normal). The positive control reduced IL-6 to 95.0 ± 8.4 pg/mL (*** *p* < 0.001 vs. model), whereas AF-SD further lowered IL-6 to 95.33 ± 7.2 pg/mL, representing a 49.56% reduction (*** *p* < 0.001 vs. model) and remaining significantly lower than the positive control (** *p* < 0.01).

Together, these results demonstrate that AF-SD effectively suppressed ulcer-induced upregulation of the pro-inflammatory cytokines TNF-α and IL-6, exhibiting a superior anti-inflammatory effect compared with the clinically used spray.

#### 2.4.2. Serum Levels of VEGF

In contrast to inflammatory cytokines, serum VEGF levels displayed an opposite trend (Figure 4C). The normal group showed a VEGF concentration of 85.68 ± 12.3 pg/mL. VEGF levels decreased slightly in the model group to 68.29 ± 10.6 pg/mL, suggesting suppression of pro-healing angiogenic signaling during ulcer-related inflammation.

In the positive control group, VEGF increased to 96.66 ± 15.8 pg/mL, representing a 41.54% increase over the model group. AF-SD produced a more pronounced elevation, raising VEGF to 129.46 ± 18.2 pg/mL (*** *p* < 0.001 vs. model), an increase of 89.57%. This marked upregulation indicates that AF-SD not only suppresses inflammation but also enhances angiogenic activity critical for tissue repair.

### 2.5. Histopathological Evaluation

#### 2.5.1. H&E Morphological Assessment

**Figure 5:**
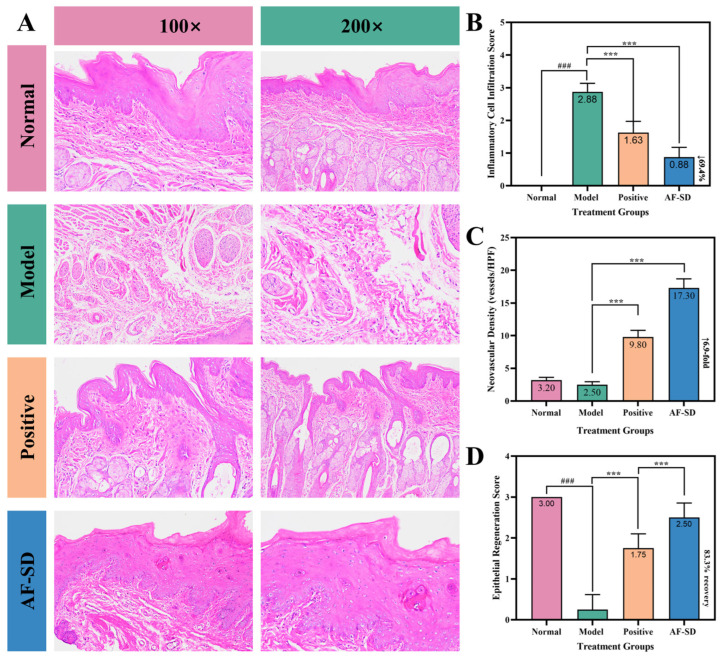
Histopathological morphology and quantitative analyses of inflammation, angiogenesis, and epithelial repair. (**A**) Representative H&E images. (**B**) Inflammatory cell infiltration scores. (**C**) Neovascular density (vessels/HPF). (**D**) Epithelial regeneration scores. *** indicates *p* < 0.001, ^###^ indicates that there is a significant difference between the control group and the control group.

H&E-stained sections of the buccal mucosa are shown in Figure 5A. In the normal group, epithelial architecture was intact, with uniform epithelial thickness (150–180 μm), well-organized keratinized, granular, spinous, and basal layers, and clear submucosal structures with normal vasculature and minimal lymphocytic presence.

In the model group, the epithelium was extensively eroded, with only a few residual basal cells at the ulcer margin. The ulcer base was densely infiltrated with inflammatory cells—primarily neutrophils and lymphocytes—distributed diffusely. Marked submucosal edema, disorganized collagen fibers, vascular congestion, and occasional hemorrhage were observed. Neovascularization was scarce, and minimal signs of tissue repair were present.

In the positive control group, ulcer size was reduced, and partial epithelial coverage appeared. However, the regenerated epithelium remained thin (60–80 μm) and structurally incomplete. Inflammatory infiltration decreased but remained moderate, with lymphocytes and mononuclear cells localized focally. Submucosal edema improved, and moderate neovascularization (8–12 vessels/HPF) was observed, though vessel lumens were small and endothelium immature.

The AF-SD group exhibited the most advanced tissue repair. Most of the ulcer area was covered by newly formed epithelium with near-normal thickness (120–140 μm). Although the keratin layer was still thin, the epithelial layers were largely restored, with orderly basal cell arrangement and visible mitotic activity. Inflammatory infiltration was markedly reduced, with only sparse lymphocytes present. Submucosal architecture approached normal, edema resolved, and collagen fibers were realigned. Notably, neovascularization was abundant (15–20 vessels/HPF), with well-defined lumens and plump endothelial cells, some containing erythrocytes, indicating functional blood flow. Active fibroblast proliferation beneath the epithelium further supported robust tissue remodeling.

#### 2.5.2. Semi-Quantitative Analysis

Semi-quantitative assessments derived from H&E-stained sections are summarized in Figure 5. Inflammatory cell infiltration scores (Figure 5B) showed a clear gradient among groups. The normal mucosa exhibited no infiltration (score 0), whereas the model group displayed severe inflammatory infiltration with a score of 2.88 ± 0.35. This score was reduced to 1.63 ± 0.52 in the positive-drug group (*** *p* < 0.001 vs. model), indicating a moderate degree of residual inflammation. Notably, AF-SD treatment further decreased the infiltration score to 0.88 ± 0.35 (*** *p* < 0.001 vs. model), which was significantly lower than that of the positive-drug group and consistent with only mild inflammatory activity.

Neovascular density (Figure 5C) followed a complementary trend. Baseline vessel density in normal mucosa was 3.2 ± 0.8 vessels/HPF. The model group showed a slight but non-significant reduction to 2.5 ± 0.7 vessels/HPF (*p* > 0.05). Treatment with the positive drug increased neovascularization to 9.8 ± 1.5 vessels/HPF (*** *p* < 0.001 vs. model). AF-SD produced a markedly greater effect, elevating vessel density to 17.3 ± 2.1 vessels/HPF (*** *p* < 0.001 vs. model), a 6.9-fold increase relative to the model group and significantly higher than the positive-drug group (*** *p* < 0.001).

Epithelial regeneration scores (Figure 5D) exhibited a similar hierarchy. Normal mucosa scored 3, whereas the model group showed extensive epithelial loss with a score of 0.25 ± 0.46. Partial epithelial restoration was observed in the positive-drug group, which scored 1.75 ± 0.46 (*** *p* < 0.001 vs. model). AF-SD achieved the most complete epithelial repair, yielding a score of 2.50 ± 0.53 (** *p* < 0.001 vs. model) and a significantly higher level of regeneration than the positive-drug group.

Collectively, these semi-quantitative histological findings corroborate the therapeutic profile of AF-SD, demonstrating its capacity to attenuate inflammatory responses, enhance neovascularization, and facilitate epithelial regeneration. These results are fully consistent with the reductions in ulcer area and the modulation of serum biomarkers observed in parallel assays.

## 3. Discussion

### 3.1. Summary of Key Findings

In this study, an AF-SD formulation was successfully developed and its therapeutic efficacy and mechanism of action in ROU were systematically evaluated. The optimized preparation process—drug-to-carrier ratio of 1:2, melting temperature of 70 °C, and melting time of 5 min—yielded AF-SD with a 2.5-fold increase in cumulative L-borneol release at 25 min compared with raw Ai-Fen. DSC, XRD, SEM, and FTIR analyses confirmed that Ai-Fen was fully converted into an amorphous form and molecularly dispersed in the PEG 6000 matrix via hydrogen-bond interactions.

In a chemically induced rat model of ROU, AF-SD markedly accelerated ulcer healing, achieving a 74.16% healing rate by day 5, with efficacy superior or comparable to the positive control, Jinhoujian Spray. Mechanistically, AF-SD reduced serum TNF-α and IL-6 levels by 55.5% and 49.56%, respectively, while increasing VEGF by 89.57%. These dual effects—suppression of inflammation and promotion of angiogenesis—contributed to effective ulcer resolution. Collectively, the findings provide a scientific basis for the clinical application of Ai-Fen and offer a practical formulation strategy for modernizing traditional herbal medicines.

### 3.2. Mechanisms by Which Solid Dispersion Technology Enhances Ai-Fen Bioavailability

Solid dispersion technology is a classical approach to improving the bioavailability of poorly water-soluble drugs. Its core mechanisms include drug amorphization, particle size reduction, increased surface area, and improved wettability by hydrophilic carriers [26,27]. In the present work, DSC and XRD clearly demonstrated that Ai-Fen in AF-SD was completely converted from a crystalline to an amorphous state, which is the fundamental driver of the marked increase in dissolution. Amorphous drugs are not constrained by lattice energy, resulting in lower free energy barriers for dissolution and potentially several- to tens-fold higher solubility [41]. SEM further showed that the original crystalline particles of Ai-Fen disappeared in AF-SD, where the drug was highly dispersed at the molecular or nanoscopic level within the PEG matrix. This greatly increased the effective surface area in contact with the dissolution medium, consistent with the Noyes–Whitney equation, which predicts a direct proportionality between dissolution rate and surface area [42].

FTIR analysis revealed hydrogen-bond interactions—evidenced by a 13 cm^−1^ red shift in the O-H stretching band—which are crucial for stabilizing the amorphous form. Previous studies have shown that hydrogen bonding between drug molecules and polymer carriers effectively inhibits recrystallization during storage, providing a “molecular immobilization” effect that underpins the long-term stability of PEG-based solid dispersions [43,44]. In this study, hydroxyl and carbonyl groups in Ai-Fen likely acted as hydrogen bond donors/acceptors, forming a stable hydrogen-bond network with ether oxygen atoms in PEG, thereby locking the drug in an amorphous state. This is consistent with observations made by Leuner et al. in indomethacin solid dispersions [45].

In addition, PEG 6000 exhibits surfactant-like behavior and can reduce interfacial tension between Ai-Fen and water, improving wettability [46]. We observed that AF-SD rapidly disintegrated in the dissolution medium to form a uniform milky suspension, whereas raw Ai-Fen remained floating on the surface for an extended period. This visual difference directly illustrates the carrier’s contribution to improved wetting. Conclusively, the observed 2.5-fold enhancement in L-borneol release is directly attributed to the synergy of three factors: the thermodynamic advantage of the amorphous state (confirmed by DSC/PXRD), the molecular stabilization provided by hydrogen bonding (revealed by FTIR), and the improved wettability and dispersibility facilitated by the hydrophilic PEG 6000 carrier. This provides a robust pharmaceutic foundation for achieving rapid and effective local drug action following intraoral administration.

### 3.3. Dual Pharmacological Mechanisms of AF-SD

This study revealed a dual pharmacological mechanism by which AF-SD treats ROU: (i) suppression of pro-inflammatory cytokines and (ii) promotion of angiogenesis and tissue repair via VEGF upregulation. This combined “anti-inflammatory plus pro-repair” strategy offers a new therapeutic paradigm for ROU.

On the anti-inflammatory side, AF-SD significantly reduced serum TNF-α and IL-6 levels by 55.5% and 49.56%, respectively. TNF-α and IL-6 are key mediators in ROU pathogenesis; they amplify inflammatory cascades via NF-κB activation, leading to epithelial apoptosis and tissue damage [47,48]. L-borneol, the principal active constituent of AF-SD, has been reported to inhibit LPS-induced TNF-α and IL-6 secretion in macrophages, likely via the NF-κB signaling pathway. Existing literature suggests that L-borneol inhibits the phosphorylation and degradation of IκB-α, thereby blocking the nuclear translocation of the NF-κB p65 subunit [49,50]. This transcriptional suppression directly leads to the observed downregulation of downstream pro-inflammatory mediators verified in our ELISA results. Consistent with these reports, histopathological analyses in this study showed a marked reduction in inflammatory cell infiltration in the AF-SD group (score 0.88 ± 0.35), with a shift from neutrophil-dominant acute inflammation to a milder lymphocyte/monocyte pattern, indicating effective control of acute inflammation. The superior anti-inflammatory efficacy of AF-SD compared with Jinhoujian Spray may be attributed to enhanced local bioavailability of L-borneol. The rapid dissolution of AF-SD creates a high concentration gradient at the mucosal interface, facilitating drug penetration into the ulcerated tissue where it can exert direct anti-inflammatory effects, alongside any systemic benefits.

On the pro-angiogenic side, AF-SD increased serum VEGF levels 5.92-fold, substantially exceeding the 2.92-fold elevation observed with the positive control. VEGF is a master regulator of angiogenesis; by binding to VEGFR-2 on endothelial cells, it activates PI3K/Akt and MAPK/ERK signaling pathways, thereby promoting endothelial cell proliferation, migration, and tube formation [51,52]. Adequate angiogenesis is critical for ulcer healing, not only supplying oxygen and nutrients but also delivering growth factors and immune cells to support wound debridement and granulation tissue formation [53]. In this study, AF-SD increased neovascular density to 17.3 vessels/HPF—6.9-fold higher than that in the model group—with well-formed lumens and plump endothelial cells, consistent with effective functional perfusion. The new vessels were mainly located at the ulcer base and margins, closely aligned with regions of active epithelial regeneration, fitting the canonical “vascularization–re-epithelialization” pattern of tissue repair [54].

The mechanism by which L-borneol enhances VEGF expression is not yet fully elucidated. Some studies suggest that moderate oxidative stress can induce hypoxia-inducible factor-1α (HIF-1α), thereby upregulating VEGF [55]. Although L-borneol exhibits antioxidant properties, it may, at certain concentrations, modulate redox homeostasis to indirectly activate the HIF-1α/VEGF axis [56]. Furthermore, improvement of the inflammatory microenvironment may also contribute to VEGF upregulation: by lowering TNF-α and IL-6 levels, AF-SD likely alleviates inflammation-mediated suppression of angiogenesis, thus creating a permissive environment for VEGF-driven neovascularization [57].

This dual mechanism stands in sharp contrast to traditional ROU therapies. Corticosteroids, while potently anti-inflammatory, may inhibit VEGF expression and fibroblast proliferation, thereby delaying tissue repair [58]. Jinhoujian Spray, although composed of multiple herbal components, primarily provides anti-inflammatory “heat-clearing and detoxifying” effects, with relatively weaker pro-angiogenic activity. By concurrently rebalancing inflammation and repair, AF-SD not only rapidly controls inflammation but also actively promotes tissue regeneration. This may underlie its superior efficacy compared with the positive control and suggests that, for ROU, an “anti-inflammatory only” strategy may be insufficient, whereas combined “anti-inflammatory plus pro-repair” approaches are better aligned with the physiology of wound healing.

### 3.4. Comparison with Existing Therapies for ROU

Current therapeutic options for ROU include corticosteroids, immunomodulators, and traditional Chinese medicine (TCM) formulations, each with notable limitations. Corticosteroids provide rapid symptom relief but are associated with risks of fungal infection, mucosal atrophy, and increased relapse rates with long-term use [59,60]. Immunomodulators such as thalidomide are effective in severe or refractory cases but are restricted by teratogenicity and neurotoxicity [61]. TCM sprays like Jinhoujian generally have fewer adverse effects but may act more slowly [62,63].

In this context, AF-SD demonstrated several advantages. In terms of onset, AF-SD achieved a 54.08% ulcer healing rate by day 3, significantly higher than that of the positive control, likely reflecting improved L-borneol bioavailability. Regarding comprehensiveness of efficacy, AF-SD produced a greater increase in VEGF (89.57% vs. 41.54%) and higher neovascular density (17.3 vs. 9.8 vessels/HPF), highlighting its dual “anti-inflammatory plus pro-repair” activity. Regarding the safety profile, the proposed AF-SD formulation utilizes PEG 6000 as the carrier, which is listed in the FDA’s Inactive Ingredient Database and widely recognized as a non-irritant, biocompatible excipient for mucosal application. Furthermore, Ai-Fen has been used in traditional medicine for centuries. Prior toxicological data indicate that the acute oral LD_50_ of L-borneol exceeds 3000–5000 mg/kg in rodents. In our study, the daily topical dose applied to the ulcer was approximately 2 mg of Ai-Fen, which provides a safety margin of over 1000-fold relative to the toxic threshold. Consequently, AF-SD is expected to possess a favorable safety profile for clinical use, although formal long-term irritation studies will be conducted in future phases. From a cost-effectiveness perspective, the production cost of AF-SD is approximately one-third that of equivalent-dose corticosteroid formulations and half that of Jinhoujian Spray, making it suitable for use in primary healthcare settings. However, the present chemical cauterization model primarily reflects acute ulcer healing rather than the recurrent nature of clinical ROU. Whether AF-SD can reduce ulcer recurrence requires further evaluation in chronic or relapse models and, ultimately, in clinical trials.

### 3.5. Innovation and Translational Potential

This study has three main innovative aspects: Formulation innovation. Ai-Fen was formulated as a solid dispersion for the first time, using amorphization to overcome its poor aqueous solubility. Compared with β-cyclodextrin inclusion complexes or liposomal encapsulation, solid dispersion technology is simpler, more cost-effective, and more stable, making it highly suitable for industrial-scale production [51]. Pharmacodynamic innovation: The therapeutic effects of AF-SD on ROU were systematically evaluated, and its dual mechanism—simultaneous anti-inflammatory activity and promotion of angiogenesis—was elucidated for the first time, providing new theoretical support for ROU management. Translational value: AF-SD has favorable prospects for clinical translation: raw materials are abundant (domestic Ai-Fen production reaches several hundred tons annually); the preparation process (melt-fusion) is solvent-free, simple, and technically mature, allowing for easy industrial scale-up. Furthermore, solid dispersions are highly compatible with various downstream dosage forms (e.g., gels, patches, or tablets), and the daily treatment costs are approximately 5–8 RMB, only about one-tenth the cost of imported drugs, aligning well with healthcare cost-containment policies; quality is controllable (standardized by L-borneol content); administration is convenient (topical application); and daily treatment costs are approximately 5–8 RMB, only about one-tenth the cost of imported drugs, aligning well with healthcare cost-containment policies. Moreover, the paradigm of “using solid dispersion technology to modernize traditional medicines” established here may be extended to other poorly soluble natural products such as curcumin, resveratrol, and quercetin.

### 3.6. Limitations and Future Perspectives

However, several limitations of this study should be acknowledged. First, the chemical cauterization model employed mainly mimics acute mucosal injury and does not fully capture the complex autoimmune etiology responsible for the recurrence of ROU. Second, detailed pharmacokinetic data, specifically regarding mucosal residence time and local tissue distribution, are currently lacking. Although serum cytokines confirmed systemic absorption, the local drug concentration at the ulcer site remains to be quantified. Therefore, future research directions will focus on: (1) utilizing immune-mediated relapse models to evaluate long-term efficacy; (2) performing LC-MS/MS-based PK profiling to optimize the dosing regimen; and (3) developing advanced dosage forms, such as mucoadhesive patches or buccal films incorporating AF-SD, to further prolong mucosal contact time and improve patient compliance. With these advancements, AF-SD has the potential to evolve into a safe, effective, and affordable therapeutic option for ROU.

## 4. Materials and Methods

### 4.1. Materials and Reagents

#### 4.1.1. Drugs and Carrier Materials

Ai-Fen was obtained from Guizhou Aishengyuan Pharmaceutical Co., Ltd. (Guiyang, China; batch No. AF20230615). The material was authenticated by Prof. Pang Yuxin of the School of Pharmacy, Guizhou University of Traditional Chinese Medicine, as the dried leaf extract of *Blumea balsamifera* (L.) DC. The sample was stored in a sealed desiccator protected from light at a controlled temperature of 25 ± 2 °C in the university’s Pharmaceutics Laboratory. Gas chromatography analysis confirmed that the L-borneol (IUPAC name: (1S,2R,4S)-1,7,7-trimethylbicyclo [2.2.1] heptan-2-ol; Molecular formula: C_10_H_18_O) content of this batch was 54.3 ± 2.1% (*w*/*w*), meeting the quality criteria previously established by our research group. Polyethylene glycol 6000 (PEG 6000; Structure: HO(CH_2_CH_2_O)_n_H; molecular weight 5800–6200 Da, pharmaceutical grade) was purchased from Aladdin Biochemical Technology Co., Ltd. (Shanghai, China, batch No. H2116493). The positive control drug—Jinhoujian Spray (Chinese medicine registration No. Z20025361, containing *Lonicera japonica*, *Scrophularia ningpoensis*, borneol and other herbal extracts)—was supplied by Guizhou Hongyu Pharmaceutical Co., Ltd. (Guiyang, China) [64]. Jinhoujian Spray was selected as the positive control because it is a first-line clinical TCM preparation for oral ulceration with established efficacy, and it shares borneol as a common active ingredient with Ai-Fen, allowing for a comparative evaluation of the solid dispersion’s performance.

#### 4.1.2. Reagents and Chemicals

L-borneol reference standard (purity ≥ 98%, batch No. L2116096), methyl salicylate internal standard (purity ≥ 99%, batch No. F2306774), and ethyl acetate (chromatographic grade, batch No. F2306774) were all purchased from Aladdin Biochemical Technology Co., Ltd. Sodium hydroxide (analytical grade, batch No. 20211018) was obtained from China National Pharmaceutical Group Chemical Reagent Co., Ltd. (Shanghai, China). Disodium hydrogen phosphate and potassium dihydrogen phosphate were obtained from Tianjin Guangfu Fine Chemical Research Institute (Tianjin, China) and used to prepare phosphate-buffered saline (PBS, pH 7.1). Rat TNF-α ELISA kits (batch No. ZC-37624), IL-6 ELISA kits (batch No. ZC-36404), and VEGF ELISA kits (batch No. ZC-37412) were purchased from Shanghai Zcibio Biotech Co., Ltd. (Shanghai, China). Hematoxylin-eosin (H&E) staining kits were purchased from Solarbio Science & Technology Co., Ltd. (Beijing, China). All reagents were used within their validity period. Ultrapure water (resistivity ≥ 18.2 MΩ·cm) was used throughout the experiments.

#### 4.1.3. Experimental Animals

Specific pathogen-free (SPF) female Sprague Dawley rats (200 ± 20 g, 8 weeks old) were provided by the Experimental Animal Research Center of Guizhou University of Traditional Chinese Medicine (license No. SCXK Qian 2021-0003). Animals were housed in the SPF facility under controlled environmental conditions (22 ± 2 °C, 50 ± 10% relative humidity, 12 h/12 h light–dark cycle) with free access to food and water. All animal procedures were approved by the Animal Ethics Committee of Guizhou University of Traditional Chinese Medicine (approval No. 2022125) and conducted in accordance with the Regulations on the Administration of Laboratory Animals and the NIH Guide for the Care and Use of Laboratory Animals (8th edition), with efforts made to minimize animal suffering and reduce the number of animals used.

### 4.2. Instruments and Equipment

Major instruments included: a gas chromatograph (Agilent 7890B, Agilent Technologies, Santa Clara, CA, USA) equipped with a flame ionization detector (FID) and an Agilent HP-5 quartz capillary column (30 m × 0.32 mm × 0.5 μm); a differential scanning calorimeter (DSC 25, TA Instruments, New Castle, DE, USA); a powder X-ray diffractometer (D8 Advance, Bruker, Ettlingen, Germany) equipped with a Cu Kα radiation source (λ = 1.5406 Å); a scanning electron microscope (Helios 5 CX, Thermo Fisher Scientific, Waltham, MA, USA); a Fourier-transform infrared spectrometer (TENSOR II, Bruker, Germany); a dissolution tester (RC807, Tianjin Tianguang Instrument Co., Ltd., Tianjin, China) equipped with a paddle apparatus; a microplate reader (Multiskan Sky, Thermo Fisher Scientific, USA); a high-speed centrifuge (TG16-WS, Xiangyi Laboratory Instrument Co., Ltd., Changsha, China); an electronic balance (ME204E, 0.1 mg precision, Mettler Toledo, Greifensee, Switzerland); and a digital vernier caliper (DL91150, Deli Group Co., Ltd., Ningbo, China).

### 4.3. Preparation of Ai-Fen Solid Dispersions

#### 4.3.1. Melt-Fusion Preparation Procedure

AF-SD was prepared by a melt-fusion method. Precisely weighed amounts of Ai-Fen and PEG 6000 were mixed according to the designated drug-to-carrier ratios (*w*/*w*) and transferred to a 10 mL beaker. The beaker was placed in a thermostatic water bath and heated to the target temperature until PEG 6000 was fully melted. The molten mixture was stirred gently with a glass rod to ensure uniform dispersion of Ai-Fen within the PEG melt. To minimize the loss of volatile components during heating, the beaker was sealed with polyethylene film. After the specified melting time was reached, the beaker was immediately transferred to a 4 °C refrigerator for rapid quenching for 5 min. The solidified mass was ground thoroughly for 15 min using a mortar and pestle and then passed through an 80-mesh sieve (180 μm) to obtain fine AF-SD powder, which was stored in a desiccator until use. A physical mixture (PM) of Ai-Fen and PEG 6000 was prepared as a control by blending and grinding the two components in the same proportions without melt processing.

#### 4.3.2. Single-Factor Experimental Design

To identify key formulation parameters affecting the dissolution behavior of the solid dispersions, the effects of drug-to-carrier ratio, melting temperature, and melting time on the cumulative release of L-borneol were examined.

(1) Effect of drug-to-carrier ratio

Under fixed conditions of 70 °C and 5 min, Ai-Fen and PEG 6000 were combined at mass ratios of 1:1, 1:2, 1:3, 1:4, 1:5, 1:6, 1:7, and 1:8. AF-SD samples were prepared as described in Section 2.3.1, and cumulative L-borneol release at 25 min was measured.

(2) Effect of melting temperature

At a fixed drug-to-carrier ratio of 1:2 and melting time of 5 min, melting temperatures were set at 60 °C, 70 °C, 80 °C, 90 °C, and 100 °C. The resulting AF-SD samples were evaluated for dissolution behavior.

(3) Effect of melting time

At a fixed drug-to-carrier ratio of 1:2 and melting temperature of 70 °C, melting times of 5, 6, 7, 8, 9, and 10 min were tested.

All experiments were conducted in triplicate, and mean values were reported.

#### 4.3.3. Orthogonal Design Optimization

Based on the single-factor experiments, an L_9_(3^3^) orthogonal design was employed to further optimize the preparation parameters. Three factors were selected—drug-to-carrier ratio (A), melting temperature (B), and melting time (C)—each at three levels. The cumulative L-borneol release at 25 min served as the evaluation index. The factor-level matrix is shown in Table 3, and the orthogonal array L_9_(3^3^) in Table 4. Nine AF-SD formulations were prepared accordingly, with each condition processed in triplicate. The experimental design matrix was generated, and subsequent range analysis and analysis of variance (ANOVA) were performed using IBM SPSS Statistics software (Version 26.0, IBM Corp., Armonk, NY, USA) to determine the relative importance and statistical significance of each factor, ultimately identifying the optimal preparation conditions.

### 4.4. Physicochemical Characterization

#### 4.4.1. Dissolution Testing

The dissolution behavior of L-borneol in the Ai-Fen solid dispersions was analyzed by gas chromatography (GC). The procedure followed Method II (paddle method) described in the Chinese Pharmacopoeia (2020 Edition, Part IV, General Rule 0931). Dissolution tests were performed using an automated dissolution apparatus with 900 mL of phosphate-buffered saline (PBS, pH 7.1) as the dissolution medium, maintained at 37.0 ± 0.5 °C and stirred at 70 rpm. The pH of 7.1 was selected to simulate the mean physiological environment of human saliva (typically pH 6.8–7.2) where the formulation is intended to function. Solid dispersion samples equivalent to 20 mg of Ai-Fen were accurately weighed and placed into the dissolution vessels. Aliquots of 5 mL were withdrawn at 5, 10, 15, 20, 25, and 30 min, with equal volumes of pre-warmed fresh medium added immediately to maintain sink conditions. Samples were filtered through a 0.22 μm membrane. A 1.0 mL portion of the filtrate was transferred into a 5 mL volumetric flask, followed by 1.0 mL of methyl salicylate internal standard solution (2.0 mg/mL). The mixture was diluted to volume with ethyl acetate, vortex-mixed for 2 min, allowed to separate, and the supernatant was analyzed by GC.

(1) Chromatographic conditions

HP-5 quartz capillary column (30 m × 0.32 mm × 0.5 μm); flame ionization detector (FID); nitrogen as carrier gas (1.0 mL/min); injection temperature 220 °C; detector temperature 240 °C; temperature program: 80 °C (2 min), ramp at 5 °C/min to 100 °C, then 20 °C/min to 200 °C (hold 4 min); split ratio 9:1; injection volume 0.6 μL. Under these conditions, L-borneol and the internal standard exhibited retention times of approximately 12.5 min and 15.8 min, respectively, with satisfactory resolution. High-purity nitrogen (99.999%) was used as the carrier gas at a constant flow rate of 1.0 mL/min. Nitrogen also served as the makeup gas (25 mL/min), while hydrogen (30 mL/min) and air (400 mL/min) were supplied to the FID.

(2) Calibration curve

Standard solutions of L-borneol (16.02, 24.03, 32.04, 40.05, 48.06, and 51.26 μg/mL) were mixed with equal volumes of the internal standard and analyzed. A calibration curve was constructed using the peak area ratio (A) versus L-borneol concentration (C, μg/mL). The regression equation was: A = 1050.8C–0.306 (r^2^ = 0.9998), indicating excellent linearity over the tested concentration range. The limits of detection (LOD) and quantification (LOQ) were determined to be 0.05 μg/mL and 0.15 μg/mL, based on signal-to-noise ratios of 3:1 and 10:1, respectively. Method validation showed a precision (RSD) of 0.98%, recovery of 98.56% ± 1.85% (*n* = 6), and sample stability for at least 24 h.

Dissolution profiles of raw Ai-Fen material and PM were determined using the same protocol. Each sample was tested in triplicate, and both cumulative release amount and release percentage of L-borneol were calculated.

#### 4.4.2. Differential Scanning Calorimetry (DSC)

The thermal behavior of the Ai-Fen solid dispersions was evaluated using a DSC 25 differential scanning calorimeter. Samples of Ai-Fen, PEG 6000, PM, and AF-SD (5–8 mg each) were accurately weighed and sealed in aluminum pans. An empty aluminum pan served as the reference. Measurements were performed under nitrogen flow (50 mL/min), with a heating rate of 10 °C/min from 30 °C to 300 °C. Thermograms were recorded to determine the melting temperature (Tm), melting enthalpy (ΔHm), and glass transition temperature (Tg), which were used to assess changes in the physical state of Ai-Fen within the solid dispersion matrix. Each sample was analyzed in duplicate.

#### 4.4.3. Powder X-Ray Diffraction (PXRD)

The crystalline characteristics of the samples were examined using a powder X-ray diffractometer. Sample powders were uniformly spread onto the sample holder and gently pressed to obtain a flat surface. Diffraction patterns were recorded using Cu Kα radiation (λ = 1.5406 Å) at 40 kV and 40 mA. The scanning range was 2θ = 5–60°, with a scan rate of 5°/min and a step size of 0.02°. PXRD profiles of Ai-Fen, PEG 6000, PM, and AF-SD were compared to assess changes in crystallinity and drug dispersion state within the carrier.

#### 4.4.4. Scanning Electron Microscopy (SEM)

SEM was employed to observe surface morphology and microstructural characteristics. Small amounts of sample powder were mounted on conductive adhesive tape and sputter-coated with gold under vacuum (coating thickness~10 nm) to enhance conductivity. Images were captured at an accelerating voltage of 5 kV, working distance of 8–10 mm, and magnifications of 500×, 2000×, and 5000×. Morphological differences among Ai-Fen, PEG 6000, PM, and AF-SD were evaluated to visualize the dispersion state of the drug in the carrier.

#### 4.4.5. Fourier Transform Infrared Spectroscopy (FTIR)

FTIR analysis was conducted to investigate potential changes in functional groups and interactions between Ai-Fen and PEG 6000. Samples (~2 mg) were mixed with 200 mg of dried KBr, ground uniformly, and compressed into pellets under infrared light. A blank KBr pellet was used as background. Spectra were recorded in the range of 4000–400 cm^−1^ with a resolution of 4 cm^−1^ and 32 accumulated scans. FTIR spectra of Ai-Fen, PEG 6000, PM, and AF-SD were compared with particular attention to shifts or intensity changes in characteristic absorption bands that may indicate hydrogen bonding or other intermolecular interactions.

### 4.5. Animal Experiments

#### 4.5.1. Establishment of the ROU Model

A rat model of recurrent oral ulceration (ROU) was established using a chemical cauterization method with minor modifications from previously described protocols. After a 7-day acclimatization period, rats were fasted for 12 h before modeling but allowed free access to water. Deep anesthesia was induced by intraperitoneal injection of 10% chloral hydrate (3.5 mL/kg). Once the animals no longer responded to painful stimuli, they were positioned and fixed on an operating board. A medical cotton swab was dipped in 50% NaOH solution and gently applied to the right buccal mucosa, approximately 0.5 cm from the commissure, for 3 min to induce a circular ulcer with a diameter of about 3–4 mm. Immediately after cauterization, the lesion was thoroughly wiped three times with saline-soaked swabs (30 s each) to neutralize residual alkali and remove necrotic tissue. All procedures were performed under aseptic conditions to prevent secondary infection. At 24 h post-cauterization, ulcer formation was evaluated. Successful modeling was defined by a clearly demarcated ulcer with a yellowish-white base surrounded by hyperemic and edematous mucosa. Animals in which modeling failed or whose ulcer area deviated by more than 20% from the group mean were excluded. The final success rate was approximately 85–90%.

#### 4.5.2. Grouping and Treatment Regimen

Rats with successfully induced ulcers were randomly allocated into four groups based on ulcer area (*n* = 8 per group): Normal group: Healthy rats without ulcer induction, receiving no treatment. Model group: Ulcer-induced rats treated topically with an equal volume of normal saline at the ulcer site once daily. Positive control group: Ulcer-induced rats treated once daily with Jinhoujian Spray applied directly to the lesion (three puffs per administration, approximately 0.15 mL). AF-SD group: Ulcer-induced rats treated once daily with a suspension of Ai-Fen solid dispersion applied to the ulcer surface.

(1) Preparation of the AF-SD suspension

The optimized AF-SD formulation was dispersed in 0.5% (*w*/*v*) sodium carboxymethyl cellulose (CMC-Na) aqueous solution to obtain a suspension containing 20 mg/mL Ai-Fen. The suspension was sonicated for 5 min immediately before use to ensure uniform dispersion.

(2) Administration protocol

Topical treatment was initiated 24 h after model establishment and continued once daily for 5 consecutive days. During each treatment, a medical cotton swab was used to apply 0.1 mL of the test preparation (equivalent to 2 mg Ai-Fen) gently onto the ulcer surface. The preparation was left in contact with the lesion for approximately 30 s before the animal was allowed to swallow spontaneously. Food and water were withheld for 30 min before and after each administration to prolong drug contact with the ulcer. Before each daily treatment, the major (a) and minor (b) diameters of the ulcer were measured using a vernier caliper, and the ulcer area (S, mm^2^) was calculated according to the ellipse formula:

(1) $$S=\frac{\pi \times a\times b}{4}$$

Macroscopic images of the ulcers were also captured. Throughout the treatment period, general conditions (body weight, activity, food intake) and local ulcer characteristics (color, exudation, and bleeding) were monitored and recorded.

#### 4.5.3. Ulcer Area Measurement

Ulcer area was quantified using a digital vernier caliper. Prior to each daily administration, measurements were performed under natural light. The longest diameter (a) and the shortest diameter perpendicular to it (b) were measured with a caliper of 0.01 mm accuracy. Each dimension was measured three times, and the mean value was used to calculate ulcer area using the elliptical formula:

(2) $$S=\frac{\pi \times a\times b}{4}({\mathrm{mm}}^{2})$$

To minimize measurement bias, all assessments were conducted by the same trained investigator under consistent lighting conditions. In parallel, standardized photographs of the ulcers were taken using a digital camera (Canon EOS 850D, Tokyo, Japan, macro lens) at a fixed distance (20 cm) and angle, with a ruler included in the field as a reference scale. The ulcer healing rate (%) for each rat was calculated as:

(3) $$\mathrm{Healing}\;\mathrm{rate}=\frac{S_{0}\;-\;S_{t}}{S_{0}}\times 100\%$$

where S_0_ is the ulcer area before treatment, and S_t_ is the ulcer area on day t of treatment.

### 4.6. Biological Evaluation

#### 4.6.1. Serum Collection

At the end of the treatment period (day 6), rats were fasted for 12 h with free access to water and then deeply anesthetized with 10% chloral hydrate (3.5 mL/kg, intraperitoneally). After confirming deep anesthesia, the animals were placed in the supine position and fixed. A midline incision was made to open the abdominal cavity and expose the abdominal aorta. Approximately 5 mL of whole blood was collected from the abdominal aorta using a sterile disposable syringe and transferred into plain centrifuge tubes without anticoagulant. The blood was allowed to clot at room temperature for 30 min and then centrifuged at 3000 rpm for 15 min at 4 °C. The supernatant (pale yellow serum) was carefully separated, aliquoted into sterile Eppendorf tubes, and immediately stored at −80 °C for subsequent analysis of inflammatory and angiogenic factors. Following blood collection, the rats were euthanized by cervical dislocation. Buccal mucosal tissues containing the ulcer area were rapidly excised and gently rinsed with pre-chilled saline to remove surface blood and debris, then blotted dry with filter paper. The tissue was divided into two portions: one was snap-frozen in liquid nitrogen and stored at −80 °C for potential further analyses, and the other was fixed in 10% neutral buffered formalin for at least 24 h for histopathological examination.

#### 4.6.2. Measurement of Inflammatory Cytokines (ELISA)

Serum levels of TNF-α, IL-6, and VEGF were quantified using commercial ELISA kits according to the manufacturers’ instructions. Frozen serum samples were thawed at room temperature and diluted to the appropriate ratios recommended in the kit manuals (1:2 for TNF-α and IL-6; 1:5 for VEGF). A double-antibody sandwich ELISA format was employed. Each sample was tested in triplicate wells, along with standard and blank wells. After completing the incubation and washing steps, absorbance at 450 nm was measured using a microplate reader, with 630 nm as the reference wavelength. Standard curves were generated for each assay, and cytokine concentrations in samples were calculated accordingly. Only assays with a standard curve correlation coefficient r^2^ ≥ 0.99 and intra-assay coefficient of variation (CV) < 10% were accepted. Data are expressed as mean ± standard deviation (Mean ± SD, pg/mL).

#### 4.6.3. Histopathological Analysis (H&E Staining)

Oral mucosal tissues fixed in 10% neutral buffered formalin for at least 24 h were rinsed under running water and processed by standard histological procedures, including dehydration, clearing, paraffin infiltration, and embedding. Paraffin blocks were sectioned into 5 μm-thick serial sections using a rotary microtome and baked at 60 °C for 2 h. After deparaffinization and rehydration, sections were stained with hematoxylin and eosin (H&E) following the instructions of the staining kit, and mounted with neutral resin.

Histological changes in the ulcerated mucosa—including epithelial regeneration, inflammatory cell infiltration, neovascularization, and the structure of the submucosal tissue—were examined under a light microscope. For each sample, five non-overlapping high-power fields (×400) were randomly selected for semi-quantitative assessment. Three parameters were evaluated: (1) inflammatory cell infiltration (scored 0–3), with 0 indicating absence of inflammatory cells, 1 indicating sparse scattered infiltration, 2 indicating moderate focal aggregation, and 3 indicating extensive diffuse infiltration; (2) neovascularization, quantified as the number of newly formed vessels with clearly defined lumens per high-power field (HPF); and (3) epithelial regeneration (scored 0–3), with 0 representing no epithelial coverage, 1 representing incomplete epithelialization, 2 indicating nearly complete but thinned epithelium, and 3 indicating fully restored and normally thickened epithelium. All evaluations were independently performed by two blinded investigators, and the mean values were used for statistical analysis. Inter-observer agreement was assessed using Cohen’s kappa coefficient, with Kappa > 0.75 considered indicative of good consistency.

### 4.7. Statistical Analysis

All data were analyzed using SPSS 26.0 (IBM Corporation, Armonk, NY, USA) and GraphPad Prism 9.0 (GraphPad Software, San Diego, CA, USA). Quantitative data are presented as mean ± standard deviation (Mean ± SD). Prior to statistical testing, data distribution was evaluated using the Shapiro–Wilk test for normality, and homogeneity of variance was assessed by Levene’s test. For normally distributed data with homogeneous variances, one-way analysis of variance (one-way ANOVA) was performed for multiple-group comparisons, followed by Tukey’s honestly significant difference (HSD) post hoc test for pairwise comparisons. For data that did not meet the assumptions of normality or homogeneity of variance, the Kruskal–Wallis H test was used, with Dunn’s multiple comparison test as the post hoc procedure. For the orthogonal design experiments, range analysis and ANOVA were applied to evaluate the magnitude and statistical significance of factor effects. Inter-rater reliability of histopathological scoring was assessed using Cohen’s kappa coefficient. A *p*-value < 0.05 was considered statistically significant, and significance levels were denoted as *p* < 0.05, *p* < 0.01, and *p* < 0.001. All experiments were independently repeated at least three times, and each animal group contained eight rats (*n* = 8).

## 5. Conclusions

In this study, an Ai-Fen solid dispersion (AF-SD) was successfully developed, and its preparation was optimized using an L_9_(3^3^) orthogonal design. The optimal formulation—drug-to-carrier ratio of 1:2, melting temperature of 70 °C, and melting time of 5 min—resulted in a 2.5-fold increase in the cumulative dissolution of L-borneol at 25 min compared with the raw material. DSC, XRD, SEM, and FTIR analyses confirmed that Ai-Fen was predominantly transformed into an amorphous state within the PEG 6000 matrix, where hydrogen-bond interactions (indicated by a 13 cm^−1^ red shift in the O-H peak) contributed to the formation of a stable molecular dispersion system and effectively inhibited recrystallization. In a chemically induced rat model of recurrent oral ulceration, AF-SD markedly accelerated ulcer healing, achieving a 74.16% healing rate on day 5. Mechanistic experiments demonstrated that AF-SD exerted a dual therapeutic action by suppressing pro-inflammatory cytokines (TNF-α reduced by 55.5%; IL-6 reduced by 49.56%) while simultaneously upregulating the angiogenic factor VEGF (increased by 89.57%). This coordinated modulation of inflammation and tissue repair highlights a balanced “anti-inflammatory and pro-regenerative” treatment strategy. Overall, this study provides a solid scientific basis for the clinical application of Ai-Fen and establishes a generalizable technological framework for modernizing poorly soluble natural products. Future work should include evaluation in chronic relapse models, deeper elucidation of molecular mechanisms, and rigorous clinical trials to validate long-term efficacy, safety, and effects on recurrence.

## Figures and Tables

**Table 1:** Results of the L_9_(3^3^) Orthogonal Design.

Experiment No.	A: Ratio	B: Temp (°C)	C: Time (min)	Release (mg)	Rank
1	1 (1:2)	1 (70)	1 (5)	32.08 ± 1.52	1
2	1 (1:2)	2 (85)	2 (6)	30.57 ± 1.38	2
3	1 (1:2)	3 (100)	3 (7)	28.75 ± 1.45	5
4	2 (1:4)	1 (70)	2 (6)	29.86 ± 1.62	3
5	2 (1:4)	2 (85)	3 (7)	28.14 ± 1.55	6
6	2 (1:4)	3 (100)	1 (5)	27.23 ± 1.48	8
7	3 (1:6)	1 (70)	3 (7)	29.52 ± 1.58	4
8	3 (1:6)	2 (85)	1 (5)	27.68 ± 1.51	7
9	3 (1:6)	3 (100)	2 (6)	26.95 ± 1.42	9
K1	91.4	91.46	86.99	87.17	-
K2	85.23	86.39	87.38	87.32	-
K3	84.15	82.93	86.41	86.29	-
$\bar{k1}$	30.47	30.49	29	29.06	-
$\bar{k2}$	28.41	28.8	29.13	29.11	-
$\bar{k3}$	28.05	27.64	28.8	28.76	-
Range R	2.42	2.85	0.33	0.35	-
Rank	2	1	3	-	-

**Table 2:** ANOVA for the Orthogonal Design.

Source	SS	df	MS	F	*p*-Value	Significance
A: Ratio	13.52	2	6.76	18.54	0.008 **	Significance
B: Temp	18.94	2	9.47	25.98	0.003 **	Significance
C: Time	0.28	2	0.14	0.38	0.695	Not significant
Error	1.46	4	0.365	-	-	-
Total	34.2	10				

Note: ** *p* < 0.01 indicates a highly significant difference.

**Table 3:** Factor-Level Matrix for the Orthogonal Design.

Level	Factor A: Drug-to-Carrier Ratio (*w*/*w*)	Factor B: Melting Temperature (°C)	Factor C: Melting Time (min)
1	1:2	70	5
2	1:4	85	6
3	1:6	100	7

**Table 4:** L_9_(3^3^) Orthogonal Experimental Design.

Experiment No.	A: Ratio	B: Temp (°C)	C: Time (min)
1	1 (1:2)	1 (70)	1 (5)
2	1 (1:2)	2 (85)	2 (6)
3	1 (1:2)	3 (100)	3 (7)
4	2 (1:4)	1 (70)	2 (6)
5	2 (1:4)	2 (85)	3 (7)
6	2 (1:4)	3 (100)	1 (5)
7	3 (1:6)	1 (70)	3 (7)
8	3 (1:6)	2 (85)	1 (5)
9	3 (1:6)	3 (100)	2 (6)

## Data Availability

The original contributions presented in this study are included in the article. Further inquiries can be directed to the corresponding author.

## Abbreviations

The following abbreviations are used in this manuscript:

AF	Ai-Fen
AF-SD	Ai-Fen Solid Dispersion
ROU	Recurrent Oral Ulcer
RAS	Recurrent Aphthous Stomatitis
L-borneol	Levoborneol
DSC	Differential Scanning Calorimetry
PXRD	Powder X-ray Diffraction
SEM	Scanning Electron Microscopy
FTIR	Fourier-Transform Infrared Spectroscopy
PM	Physical Mixture
PEG	Polyethylene Glycol
CMC-Na	Carboxymethylcellulose Sodium
TNF-α	Tumor Necrosis Factor-α
IL-6	Interleukin-6
VEGF	Vascular Endothelial Growth Factor
HPF	High-Power Field
H&E	Hematoxylin and Eosin
ANOVA	Analysis of Variance
PBS	Phosphate-Buffered Saline
